# Hiding in Plain Sight: Modern Thiamine Deficiency

**DOI:** 10.3390/cells10102595

**Published:** 2021-09-29

**Authors:** Chandler Marrs, Derrick Lonsdale

**Affiliations:** 1 Independent Researcher, Henderson, NV 89074, USA; 2 Emeritus, Cleveland Clinic, Cleveland, OH 44195, USA; derricklonsdale@hotmail.com

**Keywords:** thiamine deficiency, thiamine deficiency metabolic disease, thiamine deficiency hyperglycemia, thiamine deficiency critical illness

## Abstract

Thiamine or vitamin B1 is an essential, water-soluble vitamin required for mitochondrial energetics—the production of adenosine triphosphate (ATP). It is a critical and rate-limiting cofactor to multiple enzymes involved in this process, including those at the entry points and at critical junctures for the glucose, fatty acid, and amino acid pathways. It has a very short half-life, limited storage capacity, and is susceptible to degradation and depletion by a number of products that epitomize modern life, including environmental and pharmaceutical chemicals. The RDA for thiamine is 1.1–1.2 mg for adult females and males, respectively. With an average diet, even a poor one, it is not difficult to meet that daily requirement, and yet, measurable thiamine deficiency has been observed across multiple patient populations with incidence rates ranging from 20% to over 90% depending upon the study. This suggests that the RDA requirement may be insufficient to meet the demands of modern living. Inasmuch as thiamine deficiency syndromes pose great risk of chronic morbidity, and if left untreated, mortality, a more comprehensive understanding thiamine chemistry, relative to energy production, modern living, and disease, may prove useful.

## 1. Introduction

“There is often something sinister about familiar concepts. The more familiar or ‘natural’ they appear, the less we wonder what they mean; but because they are widespread and well-known, we tend to act as if we know what we mean when we use them [1].”

Classically defined thiamine deficiency (TD) disorders in the context of alcoholism and malnutrition are familiar, taught in science and health textbooks from high school onward, and yet, for all of that familiarity, not only are most severe cases of deficiency missed, but the early stages, where symptoms are most easily treated, are entirely disregarded [2]. This is likely due in part to the fact that late 19th and early 20th century descriptions, which still hold sway today, portray the TD as an outcome of starvation-based malnutrition, where emaciation is a common visual. In food secure countries, where obesity reigns, it is difficult to consider TD within this context. Symptoms also may be overlooked in countries where fortified foods comprise the majority of calories and thiamine intake is estimated to be above the recommended daily allowance (RDA). Under these circumstances, TD is considered rare. Decades of research data, discussed later in this paper, suggest that it is not and find overt deficiency in large swaths of patient populations not designated as being at risk via familiar parameters. These individuals are not underfed and they are not likely to consume less than the RDA recommended amount of thiamine. Indeed, they are more likely to be overfed, sometimes obese, and to consume sufficient thiamine based on the current RDA values. This begs many questions, not the least of which is whether we are conceptualizing nutrient deficiency too rigidly.

Insofar as thiamine status is not routinely measured in clinical care and there are no established standards for what constitutes lower or suboptimal thiamine concentrations, or even consensus on what values constitute frank deficiency [3], it is difficult to ascertain whether the existing recommendations for thiamine sufficiency are adequate to meet the demands of individuals living in modern, industrialized countries. It is conceivable that they are not and that by presuming sufficiency based solely upon estimated intake compared to RDA values or the absence of frank symptomology and laboratory confirmation, we are missing gradations of disease linked to insufficient thiamine. In light of this possibility, we review current definitions of TD and recommendations involving thiamine sufficiency and deficiency versus investigated rates of TD. We also explore the metabolic changes precipitated by insufficient thiamine and the mechanisms by which thiamine availability is degraded in food-secure countries.

## 2. Thiamine Deficiency Definitions and Testing

Below is a brief overview of current symptoms and testing parameters regarding TD.

### 2.1. Symptoms

The early symptoms of TD are non-specific and may be easily attributed to any number of disease processes. Unrelenting or uncharacteristic fatigue, changes in mood with a tendency towards hyper-irritability and mood lability are common [4]. A sense of mental fuzziness and subtle decrements in memory are often reported, along with loss of appetite, sleep disturbances and/or gastrointestinal (GI) discomfort and dysmotility. Food intolerances and vomiting may develop as the deficiency progresses. Experimental [5] and case literature [6] suggest GI discomfort and dysmotility may be more prevalent early indications of TD than currently appreciated. A form of GI beriberi has been identified but is under-recognized [7].

Due to the vagueness of early symptoms of TD, conventional descriptions of TD focus more squarely on the later stage manifestations of wet and dry beriberi and Wernicke’s (WE) and Korsakoff’s encephalopathies, categorizing the symptoms by organ involvement. Notably, the high output cardiac failure and edema is associated with wet beriberi, the peripheral neuropathies, muscle pain, and weakness are associated with dry beriberi, and the classic neurological triad of mental confusion, ocular abnormalities and ataxia are associated with WE. Symptoms of Korsakoff’s syndrome, a more severe extension WE, include confabulation, psychosis, and significant memory deficits. Again, however, absent traditional risk factors, e.g., malnutrition, alcoholism or severe gastrointestinal distress or illness [2], the similarity of these symptoms with that of other conditions, makes clinical diagnosis difficult. To that point, a post-mortem investigation of 131 cases of WE, found 80% of cases were missed while the patient was alive likely because only 16% presented with the classic triad, 44% had one or two of the three symptoms and 19% had none at all [8].

### 2.2. Testing

Owing to vagueness of TD symptoms, emphasis is placed on laboratory confirmation. This too may present problems, depending upon the test method utilized. For clinical purposes, the most important thiamine analyte is thiamine pyrophosphate (TPP), also called thiamine diphosphate (ThDP/TDP). Additional phosphates can be added or subtracted to form thiamine triphosphate (TTP/ThTP) and thiamine monophosphate (TMP/ThMP), which are detectable by different laboratory measures, but as of yet, their utility in the clinic has not been fully extrapolated [3]. It should be noted that the phosphorylation of free thiamine into TPP, requires magnesium and ATP, and so, among the factors that will affect TPP values is magnesium deficiency [9].

Thiamine may be tested from whole blood, erythrocytes, serum, plasma, and urine. From whole blood, all three derivatives of free thiamine can be obtained. Thiamine pyrophosphate accounts for almost 90% of circulating thiamine, 80% of which, is found in erythrocytes [10]. Free thiamine, TMP and TTP are found primarily in serum, plasma, and urine.

TD may be defined by various laboratory tests. The most common tests are whole blood measures of TPP using liquid chromatography-tandem mass spectrometry (LC/MS/MS). In the US, the reference ranges LC/MS/MS TPP from two major labs, Quest Diagnostics and LabCorp, are 78–185 nmol/L and 66.5−200.0 nmol/L, respectively [11,12]. Published reference intervals for whole-blood TPP vary widely across labs, however, from a lower limit of 63–105 nmol/L to an upper limit of 171–229 nmol/L [3]. Under some conditions, TMP, TTP, and total thiamine values will be reported. Whole blood measures of TPP, while convenient, are susceptible to interference with a number of variables. Notably, slight changes in hematocrit yield corresponding changes in thiamine values in well-nourished individuals. Anemia may also impact thiamine values [13].

More accurate assessments of thiamine involve those that measure TPP from erythrocytes. Whole blood samples may measure TPP directly from isolated erythrocytes, such as with high performance liquid chromatography (HPLC), or indirectly, such as in the case of the erythrocyte transketolase activation test (ETKA). Reference ranges using HPLC methods in healthy individuals, 70–180 nmol/L for TPP and 75–195 nmol/L for total thiamine [14].

The ETKA test measures both basal and thiamine stimulated activity of the thiamine dependent enzyme transketolase. Test values are reported as a ratio or percentage of enzyme activation. When thiamine concentrations are sufficient, the addition of thiamine will not activate the transketolase enzyme. When thiamine is insufficient or deficient, transketolase activity will increase proportionately to the deficiency. Higher values correspond with the severity of deficiency. Clinically, activation >15% may be considered indicative of TD. Values >25% are considered severe and those >40% are noted with WE. [15,16] Experimentally, however, the definition of deficiency varies based upon the purpose of the study. Comparison of lab tests, for example, considers activation >25% deficient [17].

Plasma/serum contain only a small fraction of circulating thiamine relative to the erythrocytes and are sensitive to recent intake. As such, tests using plasma or serum are considered less accurate diagnostically but many labs still offer these tests. The reference range for Quest is 8–30 nmol/L [18]. More commonly, plasma measures of thiamine are used for research purposes. Similarly, urinary measures of free thiamine, TMP, and other thiamine metabolites are used in research protocols involving excretion rates relative to medication [19] deficiency states [20] and/or dietary intake [21].

Insofar as both experimental and clinical testing methods and reference ranges vary, assumptions of sufficiency based upon current methodologies are problematic, especially when thiamine concentrations border TD by some methods or reference ranges but not others. Additionally, difficulties arise when considering subclinical TD, metabolic, or genetic impairments that may demand quantitatively more thiamine than would be appreciated by current testing parameters. There may be gradations of sufficiency relative to individual genetics, diet, illness profile and environment, but this has not been investigated thoroughly [3].

## 3. The RDA, Food Fortification and Thiamine Sufficiency

In the US and elsewhere, daily requirements for thiamine and other vitamins and minerals were established in the late 1930s and early 1940s. Research and governmental reports indicated that 1 mg of thiamine daily was adequate to stave off the symptoms of overt TD in an adult [22]. Some reports, however, argued that while 1 mg may be sufficient to prevent frank deficiency, 1.5–2 mg of thiamine for a 70 kg/154 lb adult per day was more optimal and provided health benefits beyond simply the prevention of deficiency [4,23]. These reports notwithstanding, 1.1–1.2 mg of thiamine per day was adopted as the RDA and has remained unchanged for 80 years.

To ensure thiamine sufficiency, the Committee on Food and Nutrition recommended that thiamine, niacin, riboflavin, and iron be added to flour beginning in 1940. This was voluntary, but adopted broadly. In 1943, the FDA issued a statement indicating that fortification, ‘contributes substantially to the nutritional well-being of the individual who consumes usual amounts of the food’ [24]. Since then, consumption of enriched (nutrients lost to processing are replaced) or fortified (adding nutrients not originally in the food) products have become the primary route to nutrient sufficiency in developed countries, particularly in the US. An analysis of the 2009–2012 National Health and Nutrition Examination Survey (NHANES) data investigating the nutrient status of Americans found that if it were not for fortified foods, vitamin and mineral deficiencies would be rampant. For thiamine specifically, absent fortified foods, 41% of the survey respondents, would not meet the estimated average requirement (EAR). With fortified foods, however, only ~5% did not meet suggested intakes [25]. EAR represents the average daily level of intake estimated to meet the requirements of 50% of healthy individuals and are slightly lower than RDA values. The EAR for thiamine is 1.0 mg/day for men and 0.9 mg/day for women [26].

With such a low RDA for thiamine and a high rate of fortified food consumption, TD is believed to be rare in developed, food-secure countries, except in certain populations or medical situations. As a result of this perception, thiamine is not consistently assessed in healthcare practice or in the nutritional surveys that guide policy. To that end, the current NIH fact sheet updated on 26 March 2021, states “no current data on rates of thiamin deficiency in the U.S. population are available [27].”

## 4. Thiamine Deficiency in the General Population

In contradistinction to the NIH statement and the NHANES reports, a large body of research dating back decades, suggests TD is neither rare nor limited to the traditionally defined populations, but that it is simply under-recognized. In these studies, depending upon the population investigated and the assays and cutoff values used to determine thiamine status, frank deficiency has been observed in 10–>90% of subjects tested. This, of course, is in spite of diets rich in thiamine fortified foods, which in some cases, exceed the RDA by a factor of four [27]. Where available, the type of testing used to determine insufficient or deficient thiamine is noted.

### 4.1. Obesity

There is a high degree of TD in obese individuals. Here, the rate of deficiency ranges from 15 to 29% when tested prior to bariatric surgery using different methodologies (serum, whole blood) [28,29,30]. After surgery, the rate of TD climbs, and with it, an increasing risk of Wernicke’s encephalopathy (WE) [31,32]. There are no data regarding deficiency in the broader population of overweight or obese subjects, but insofar as 42% of the US population was considered obese in 2018 [33] and 39% of the adult population worldwide is considered overweight or obese, the possibility of such a high percentage of TD in this population poses a significant public health problem [34].

### 4.2. Diabetes

For individuals with Type 1 or Type 2 diabetes, plasma thiamine was estimated to be ~76% lower than in non-diabetic controls in one study [35]. In another, frank deficiency was found in 98% of the study population using plasma and urine samples [36]. The mechanisms involve hyperglycemia-driven impaired uptake in the kidneys [37] along with increased clearance [35]. Not examined in these projects: comorbid obesity, other health issues, or medication use; variables that directly affect thiamine adequacy.

### 4.3. Pregnancy

From 27 to 38% of pregnant women may not consume sufficient thiamine to ward off deficiency, even with prenatal supplementation [38,39]. A 2002 study reported the vitamin profile in 563 pregnant New Jersey women at different points across the pregnancy. They found a trend towards too much folate, riboflavin, biotin and pantothenate and too little niacin, thiamine, and vitamins A, B6, B12, suggesting that prenatal vitamins neither appropriately nor sufficiently address maternal nutrient demands [38]. For women who suffer from hyperemesis, the risk of TD is likely quite high. Although quantitative data regarding the rate of TD in hyperemetic women before reaching WE appears non-existent, case studies of hyperemesis-induced WE abound. In a troubling review of 177 cases, researchers found thiamine depletion developed between 10 and 15 weeks gestation, after 6 or fewer weeks of vomiting in 47% of the cases. The number jumped to 63% after 7 weeks of vomiting. None of the cases received thiamine while suffering from hyperemesis before WE was diagnosed; 14% received IV glucose without thiamine, provoking the onset of WE those women, and maternal mortality in one case [40]. Various methodologies to determine TD were utilized.

Sufficiency standards for thiamine during pregnancy were developed 80 years ago based on an estimated increase in growth of maternal and fetal compartments, plus an estimated increase in energy requirement, relative to non-pregnant adult women [26]. They have not been updated since.

### 4.4. Psychiatry

Since TD affects brain energy metabolism and early symptoms include nonspecific changes in mood, cognition, and motivation [41], one would expect linkage between TD and psychiatric illness. Research on the prevalence of TD in psychiatric illness is sparse, however. One study, using the EKTA test, found of 30% of psychiatric patients were deficient [42]. Another, using HPLC, found lower thiamine associated with depression in older Chinese patients [43], while others have found adjuvant thiamine improved both depressive [44] and anxiety related symptoms [45]. From the case literature, TD has been reported with bipolar disorder, schizophrenia, and as one might expect, anorexia [46].

### 4.5. Elderly

For the elderly, the possibility of TD may be high, but again, these numbers vary based upon the assays and cutoff values used to define deficiency. A study of 60 community dwelling elderly found TD in 50% of the subjects tested (EKTA), despite consuming slightly greater than the recommended daily intake [47]. Other studies have found the incidence of TD in hospitalized elderly patients ranging between 20 and 40% (EKTA and HPLC) [48,49].

### 4.6. Neurocognitive and Neuromotor Diseases

Neurocognitive and neuromotor disturbances represent the final common pathways of longstanding TD. Despite different genetic origins, several lines of evidence find associations between thiamine and Alzheimer’s [50], Parkinson’s [51], and Huntington’s diseases [52], and dementia [53], but the research on deficiency and treatment is equivocal [54]. Underlying each of these, however, is altered glucose handling, which, as will become evident later, is a hallmark of insufficient thiamine.

### 4.7. In Hospitalized Patients

When reviewing research regarding hospitalized patients, one expects the increased stress of an illness, any illness, would increase the demand for thiamine. This appears to be the case. A random sample of patients entering the emergency room in the UK found that 20% were deficient in thiamine (EKTA) [55]. For patients with heart failure, where TD should be expected, but is neither tested nor treated regularly, prevalence is high, ranging from 33 to 90%, depending upon the study and hospitalization (various methods) [56,57]. A retrospective study of 36 non-alcoholic veterans with confirmed TD found 97% had two or more chronic inflammatory conditions, 83% had one or more acute inflammatory condition, and 47% were overweight or obese, pointing to inflammation as a key factor driving TD [58].

In intensive care in general, no matter the diagnosis, TD is generally missed. One study found TD in 10% of the patient population upon admission and that number increased to 20% within a few days. Whereas another study looking specifically at patients with sepsis found 70% were deficient (various methods) [59]. This suggests that while patients may not be deficient upon admission, they may become so across time, especially if sepsis develops.

## 5. What Makes Thiamine So Important

**Figure 1:**
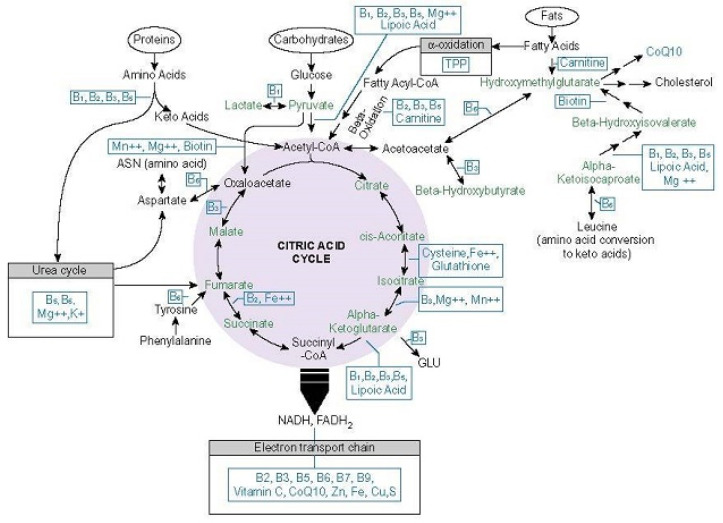
Micronutrient co-factors involved in ATP production [64]. Reprinted with permission.

By way of its necessity for enzymes at the entry points to, and at critical junctures within the mitochondria, thiamine availability dictates molecular oxygen homeostasis and mitochondrial ATP production. These two variables, then, influence the totally of organismal metabolism. Insufficient thiamine deranges mitochondrial respiration, inducing what has been termed pseudo-hypoxia [60]. Pseudo-hypoxia stabilizes hypoxia-inducible factor (HIF) proteins, which in turn, elicit a range of reactions that, while necessary to increase oxygenation acutely, become problematic when activated chronically [61]. In contrast to ischemic hypoxia, with pseudo-hypoxia although there is sufficient oxygen, the mitochondria are unable to utilize it effectively. This forces a shift towards more anaerobic metabolism and significantly reduced energy output. Here, only 2 ATP molecules are synthesized compared to the 38 ATP molecules produced via oxidative phosphorylation (OXPHOS) pathway [62]. Inadequate ATP output, further impairs oxidative capacity, initiating a range of deleterious cascades that increase vascular reactivity, inflammation, cell apoptosis, ultimately leading to organ dysfunction or failure when sufficiently severe or chronic [63]. Much of this is mediated by five key thiamine dependent enzymes or enzyme complexes: transketolase (TKT), pyruvate dehydrogenase complex (PDC), 2-hydroxyacyl-CoA lyase enzyme (HACL), branched chain keto-acid dehydrogenase (BCAKD), a-ketoglutarate dehydrogenase complex (a-KDGH). Enzymes and their nutrient co-factors involved in ATP production are illustrated below in Figure 1. Note the position of the thiamine dependent enzymes.

### 5.1. Thiamine Dependent Enzymes

#### 5.1.1. Transketolase

Thiamine is a rate-limiting factor for the TKT enzyme in the non-oxidative pentose phosphate pathway (PPP). Glucose-6-phosphate, a product derived from carbohydrate consumption, enters the PPP, where it is modified by several enzymes to produce ribose for DNA and RNA synthesis and reduced equivalents of nicotinamide adenine dinucleotide phosphate (NADPH) for steroid hydroxylation, fatty acid synthesis (myelin), and antioxidant enzymes (glutathione and thioredoxin) [65]. Ribose and NADPH are required molecules for cell functioning. TKT occurs twice in the PPP and thus becomes a critical player in this process [64]. Importantly, activities of the PPP connect to the pyruvate dehydrogenase complex (PDC), the thiamine dependent enzyme complex controlling the entry point to the oxidative branch of mitochondrial carbohydrate metabolism. In this regard, TKT function, though part of the glycolytic and non-oxidative branch of carbohydrate metabolism located in the cytosol, becomes critical to oxidative metabolism within the mitochondria.

With insufficient thiamine, TKT activity downregulates, cell proliferation, myelin synthesis, and antioxidant capacity are impaired [66] and glucose metabolism is diverted out of the PPP toward the polyol/sorbitol, hexosamine, diacylglycerol/PKC, advanced glycation end product (AGE) pathways [67]. This shift in how carbohydrates are metabolized, not only reduces substrate availability for the production of ATP, but also, becomes part of the metabolic inflexibility so commonly observed with type 2 diabetes and associated cardiovascular disease [37,68,69].

#### 5.1.2. Pyruvate Dehydrogenase Complex

A key step in carbohydrate metabolism involves the oxidative decarboxylation of pyruvate to acetyl-CoA, CO2, and NADH by the PDC enzyme complex. This step is profoundly dependent upon thiamine and as such becomes the rate-limiting variable for the oxidation of glucose by the tricarboxylic (TCA)/Krebs/citric acid cycle, but also a key player in fatty acid and cholesterol synthesis. In thiamine sufficient, steady state conditions, pyruvate is converted to acetyl CoA, which then may either enter the TCA and be further converted to oxaloacetate or be used for fatty acid or cholesterol synthesis [70]. In this regard, PDC activity becomes a central regulator of TCA function and lipid biosynthesis. Without thiamine, PDC activity diminishes and both oxidative capacity and lipid biosynthesis falter. Pyruvate, not converted to acetyl CoA, is shunted through the lactate dehydrogenase enzyme (LDH), effectively increasing lactate output and lactic acid [71]. Research in the sixties found the activity and expression of LDH isoforms were highly influenced by thiamine availability [72,73], perhaps compensatory reactions to the changing ratio of pyruvate to lactate and NADH/NAD. Additionally, pyruvate metabolism is a critical player in oxygen homeostasis via its interactions with HIF proteins. Excess pyruvate activates and stabilizes HIF1a proteins. This, in turn, not only activates the hypoxia cascades, increasing reactive oxygen species (ROS), but also, increases pyruvate dehydrogenase kinase activity (PDK), the PDC breakdown enzymes [74] further imperiling TCA function and ATP output.

#### 5.1.3. 2-Hydroxyacyl-CoA Lyase

Fatty acid metabolism also yields acetyl-CoA for entry into the OXPHOS pathway. Before reaching the mitochondria, the metabolism of fatty acids begins with alpha-oxidation in the peroxisome with HACL. HACL is thiamine dependent as well [75]. Here, food sources high in phytanic acid like beef, lamb, and products containing the cow, sheep or goat milk fats [76] or high in sphingolipids like meats, eggs, and dairy [77], are broken down before proceeding to beta-oxidation for catabolism into acetyl-CoA and subsequent entry into the TCA cycle. Elevated phytanic acid is pathognomonic for Refsum’s disease and other peroxisomal disorders, whose symptoms include: the progressive restriction of the visual field, peripheral polyneuropathy, and ataxia [78]. While disrupted sphingolipid homeostasis is associated with peripheral and central dyslipidemia and insulin resistance [79]. Combined, the symptoms closely resemble those of beriberi and the general metabolic dysfunction affecting broad swaths of the population.

#### 5.1.4. Branched Chain Keto-Acid Dehydrogenase

The metabolism of branched chain amino acids (BCAA), valine, leucine, and isoleucine, are also dependent upon thiamine via the BCKAD enzyme. With insufficient thiamine, BCAA catabolism is impaired resulting in increase in branched chain keto acids, particularly short and medium chain acylcarnitines [80]. Surplus acylcarnitines increase the flux of fatty acids through the b-oxidation pathway beyond its capacity. This results in incomplete fatty acid metabolism, e.g., dyslipidemia, and the formation of the pro-inflammatory diacylglycerol and ceramides, which again, are common findings with metabolic dysfunction [81,82].

#### 5.1.5. Alpha-Ketoglutarate Dehydrogenase Complex

Once inside the TCA cycle, thiamine availability directly influences the enzyme complex a-KDGH. The a-KDGH complex is the 4th of the TCA cycle catalyzing the conversion of a-ketoglutarate to succinyl-CoA and generating reduced nicotinamide adenine dinucleotide (NADH). Alpha-ketoglutarate concentration affects the oxidative metabolism of carbohydrates and fatty acids at various junctions (isocitrate dehydrogenase, glutamate-oxalacetic transaminase and glutamate dehydrogenase), making a-KDGH a primary site of control for metabolic flux through the TCA. A-KDGH is regulated by a complex set of variables, many of which involve thiamine availability. It is inhibited by its end products succinyl-CoA and NADH, a high ratio of ATP/ADP [83], and high, but not low, Ca^2+^ [84], and both high and low Mg^2+^ depending upon Ca^2+^ and thiamine status. Notably, Mg^2+^, in the absence of sufficient thiamine, inactivates the enzyme, while Ca^2+^ stimulates enzyme’s activity [85]. Additionally, a-KDGH is both responsible for and responsive to reactive oxidant species (ROS) production [86]. Although ROS are a natural byproduct of ATP production and serve as useful mitochondrial signaling agents, elevated ROS, relative to diminished antioxidant capacity, creates oxidative stress, damaging cellular lipids, proteins and DNA [87]. With TD, antioxidant capacity is decreased while ROS are increased [88]. This is due to changes in a-KDGH activity but also via reduced TKT activity, which supplies NADPH for glutathione [89].

#### 5.1.6. Thiamine-Influenced Enzymes

Finally, in addition to its rate-limiting role in the aforementioned enzymes, thiamine allosterically regulates the expression and activity of other enzymes within the TCA cycle. Succinate thiokinase/succinyl-CoA synthetase, which together with a-KDGH catalyzes succinyl-CoA to succinate, is reduced by 24%. Succinate dehydrogenase which oxidizes succinate to fumarate, using the electrons generated to catalyze reduction in ubiquinone to ubiquinol for complex II, thus providing the linkages between the tricarboxylic acid cycle (TCA) and electron transport chain (ETC), is reduced by 27% [90].

Decreased thiamine availability, whether absolute or relative to demand, effectively downregulates mitochondrial ATP production, while upregulating hypoxia factors, ROS, and the other toxic byproducts generated when macronutrients are diverted from thiamine dependent oxidative and non-oxidative metabolism towards alternative pathways.

## 6. Thiamine Consumption, Uptake, Activation, and Excretion

### 6.1. Consumption

The highest sources of thiamine in whole, unadulterated foods come from pork, fish (salmon, trout, tuna, catfish), many nuts and seeds (macadamia, pistachios, sunflower seeds, flax seed), beans (navy, black, black-eyed peas, lentils), peas, tofu, brown rice, whole wheat, acorn squash, asparagus, and many other foods. A diet rich in organic, whole foods should be sufficient to meet the daily requirements for the thiamine and other vitamins and minerals, absent illness states limiting absorption or increasing metabolism or excretion and absent excessive exposure to dietary, environmental or pharmaceutical anti-thiamine factors. Organic, whole foods comprise only about 4% of total food sales in the US [91]. More than 77% of the American diet consists of moderately (15.95%) or highly (61%) processed foods [92]. A diet of processed foods, however, while certainly not healthy, will meet the RDA for thiamine quite easily, perhaps even exceed it. A single serving of breakfast cereal or a few slices of bread or other fortified grain-based products is sufficient to reach the RDA for thiamine [27]. Somewhere in between the organic, whole food diet and the heavily processed diets are a range of healthier diets that also may limit thiamine intake. Fortified grains and meat contain the highest thiamine values in foods. Individuals observing grain, gluten free [93], or vegetarian [94] diets or combinations thereof may also be at risk for thiamine and other B vitamin deficiencies.

### 6.2. Uptake

Once consumed, free thiamine has to be absorbed, activated, and carried to the various tissues and organs in order to be used by the mitochondria to produce ATP. Barring dysbiosis, infection, or other intestinal challenges, consumed thiamine is absorbed in the jejunum. At high concentrations, thiamine is absorbed via passive diffusion, whereas at low concentrations, various transporters mediate uptake [41]. There are two primary thiamine transporters, ThTR1 and ThTR2, and a number of additional transporters that fall under the solute carrier family of genes:

- SLC19A1: folate transporter, but also, transports thiamine mono- and di- phospho derivatives [95].
- SLC19A2 (ThTr1): systemic thiamine transport, main transporter in pancreatic islet tissue and hematopoietic cells [96]; most abundant, from highest to lowest in the intestine, skeletal muscle, nervous system, eye, placenta, liver, and kidney [41].
- SLC19A3 (ThTr2): primary intestinal thiamine transporter [95] also located in adipose tissue, breast tissue, liver, lymphocytes, spleen, gallbladder, placenta, pancreas, and brain [41].
- SLC22A1 (OCT1): organic cation transporter 1, primary hepatic thiamine transporter [97].
- SLC25A19 (MTPP-1): mitochondrial thiamine pyrophosphate carrier [98].
- SLC35F3: endoplasmic reticulum and Golgi thiamine transporter, implicated in cardiovascular disease [99,100].
- SLC44A4 (hTPPT/TPPT-1): absorption of microbiota-generated thiamine pyrophosphate in the large intestine [101].

Transporter activity may be modified genetically [102], epigenetically [103], and via common pharmaceuticals [96]. When combined with other thiamine diminishing variables, TD may emerge. Likewise, decrements in transporter activity may be overcome with thiamine supplementation at supra-physiological doses [104,105,106,107].

### 6.3. Activation

Once absorbed, free thiamine is phosphorylated into its active form TPP. The enzyme thiamine pyrophosphokinase (thiamine diphosphokinase), catalyzes this reaction. Thiamine pyrophosphokinase is magnesium and ATP dependent. Magnesium deficiency is common in developed countries [108] and may lead to a functional TD despite sufficient thiamine [109]. For this reason, magnesium should be given with thiamine. Phosphate groups may be added and subtracted to form thiamine monophosphate or thiamine triphosphate.

While dietary thiamine is the primary factor in thiamine sufficiency, just over 2% of TPP is synthesized endogenously by various commensal bacterial populations in both the small and large intestines [110]. In the large intestine, at least 10 species of bacteria synthesize thiamine that is then directly absorbed via a population of TTP transporters (TPPT-1) in the apical membrane and are transported directly into colonocyte mitochondria via the hTPPT/TPPT-1 for ATP production [111,112]. A reduction in colonocyte thiamine, and thus ATP, would be expected to force a shift towards the more pathogenic microbial populations [113] that thrive in nutrient deficient environments [114] and dysregulate bowel motility. Local thiamine deficiency, either alone, or in combination with systemic deficiency, may contribute to small and large bowel microbial virulence and the dysmotility syndromes frequent in modern medical practice [115,116]. Adenosine thiamine triphosphate (AThTP) and adenosine thiamine diphosphate (AThDP), represent the end products of energy metabolism via a salvage pathway common to many pathogenic microbes when under duress or starvation, have been found in most mammalian tissues [117,118].

### 6.4. Storage and Elimination

Thiamine has short half-life ranging from 1 to 12 h [119]. Approximately, 30 mg of thiamine are stored in tissues with high metabolic need such as skeletal muscle, the heart, brain, liver, and kidneys. Excess free thiamine and TMP are excreted in urine [120]. Absent regular consumption, thiamine storage is depleted within 2–3 weeks. With acute illness, TD may emerge within 72 h [121].

## 7. Factors Affecting Thiamine Availability and Demand

Achieving thiamine sufficiency requires regular consumption, proper absorption and uptake, phosphorylation of free thiamine into the active thiamine compound, and adequate storage capacity. Each of these functions are impacted by a complex network of interacting reactions involving both the source of thiamine, e.g., diet, and the health, activity level, and medication usage of the individual. The regular consumption of highly processed, high calorie foods, while technically thiamine sufficient, carries an added burden of increased sugars, hydrogenated fats, and a litany of chemical additives that directly challenge thiamine metabolism and/or increase the need for thiamine beyond the recommend values. Against the backdrop of latent genetic or epigenetic factors, dysbiosis syndromes, medication use, illness, or even simply an increased activity level of the individual, maintaining adequate thiamine may be more difficult than suggested by the current understanding.

### 7.1. High Carbohydrate Diets

Perhaps the most commonly disregarded factor when considering thiamine status, is the composition of the individual’s diet. High carbohydrate diets effectively decrease circulating thiamine concentrations by a number of mechanisms. Metabolizing carbohydrates, regardless of their source or quality, diminishes thiamine stores. One study found, that when 55% of total caloric intake came from carbohydrates, no matter their source, thiamine status in otherwise healthy and thiamine sufficient individuals declined. As carbohydrate intake increased, thiamine decreased further [122]. In contrast, a lower carbohydrate, higher fat diet slows thiamine loss in thiamine-restricted experimental conditions [123] while protein seems to preserve thiamine degradation in foods [124]. Intravenous glucose or dextrose may precipitate WE in traditionally malnourished [125], hyperemetic [126] and non-traditionally malnourished, e.g., well-fed, but under nourished individuals [127].

Metabolically, high carbohydrate diets, especially those composed of highly processed, high sugar-added foods [128], are associated with hyperglycemia [129], along with up to 80% of the comorbid cardiovascular disease [130], a good percentage of neurocognitive disorders [131], and the general metabolic ill-health that plagues western countries [132]. Hyperglycemia, in turn, is frequently associated with nutrient deficiency and metabolic dysfunction affecting not just glucose handling, but fatty acid and amino acid handling as well. With hyperglycemia, the metabolism of excess sugars, those that cannot enter OXPHOS or the PPP, are diverted through the polyol/sorbitol, hexosamine, diacylglycerol/PKC, AGE pathways [67], leading to both decrements in ATP production and macro- and microvascular cell damage [69,133]. This is in addition to poor BCAA catabolism [134] with increased branched chain keto acids [80], and poor fatty acid metabolism with increased phytanic acid and disrupted sphingolipid homeostasis [81,82]. In the heart, ATP production shifts from preferred fatty acid oxidation pathway towards anaerobic glycolysis, a tale tell marker of metabolic inflexibility associated with heart failure [135], but also, wet beriberi [136]. In the brain, this pattern of metabolic dysfunction has been linked Alzheimer’s, Parkinson’s, Huntington’s and amyotrophic lateral sclerosis [137] and the symptoms of dry beriberi and WE, depending upon chronicity and severity. Each of these patterns, and the symptoms that manifest, is modulated by thiamine status relative to carbohydrate intake [136].

Both in vitro and in vivo studies demonstrate, thiamine supplementation reduces or reverses the metabolic patterns and clinical manifestations of hyperglycemia, hypertension, dyslipidemia and other associated symptoms via the upregulation of TKT, PDC and other thiamine dependent enzymes. Table 1 includes a brief sample of this research.

### 7.2. Food Chemicals

In addition to the carbohydrate load, processed foods tend carry a much higher toxicant load than unprocessed and organic foods [148]. Every aspect of commercial food production involves the usage of chemical products that are toxic to the mitochondria [145,149,150]. Many of the chemicals used in commercial agriculture [151,152,153,154] through the various channels of processing, perseveration and presentation, degrade thiamine and other nutrients when consumed [155]. The combination of high sugar, high toxic load and low thiamine and nutritional value in general, are likely at the root of much of the metabolic dysfunction affecting western populations.

### 7.3. Alcohol, Tobacco, Coffee and Tea Consumption

While chronic alcoholism is a recognized contributor to thiamine deficiency, the role of regular alcohol consumption, below the threshold of alcoholism, is underappreciated. Regardless of amount, the ethanol in alcohol blocks conversion of dietary thiamine into active thiamine [156], reducing thiamine availability by as much as 54% [157]. It is simply a matter of degree relative to chronicity that determines the rate of thiamine depletion. When consumed regularly, alcohol damages intestinal mucosa [158], resulting in impaired absorption and dysbiosis [159]. Dysbiosis, may be a cause or consequence of reduced thiamine, at least initially, but ultimately, becomes self-reinforcing if thiamine status is not corrected [112,116].

Nicotine in tobacco products, also inhibits thiamine availability via antagonism of a thiamine transporter in the pancreatic acinar cells by >40% [160] and possibly in other tissues as well. This impairs insulin secretion [161]. Nicotine use, in combination with alcohol ingestion, is implicated the development of pancreatitis [162]. Inasmuch as both limit thiamine uptake, it is conceivable pancreatitis is an indirect manifestation of thiamine deficiency.

Finally, caffeic acid, chlorogenic acid, and tannic acid in coffee, tea, and energy drinks, oxidize the thiazole ring of the thiamine molecule, impairing its absorption, while the added sugars, flavors and other substances to enhance taste, increase thiamine demand. Sixty-two percent of Americans consume an average of three cups of coffee per day [163], suggesting this popular food item may contribute more to TD than acknowledged.

### 7.4. Medications and Environmental Exposures

After diet, the next most common threat to thiamine sufficiency is the use of pharmaceuticals. Pharmaceuticals deplete thiamine and other nutrients, directly and indirectly, by a number of mechanisms [164]. Some of this is by design, such as with antibiotics that target thiamine [165] and some of it represents off-target effects, such as the blockade of thiamine transporters by metformin and the 146 other drugs tested for this action [166]. Regardless of the intended purpose, however, pharmaceuticals represent chemical impediments to thiamine and nutrient stability. As such, their regular use necessitates a concerted approach to maintain nutrient status. Among the greatest threats to thiamine status include: metformin [167], psychiatric medications [168], metronidazole [169], trimethoprim [170] and other antibiotics and anti-hypertensives [171], NSAIDs, acetaminophen, and aspirin [172], proton pump inhibitors [173], diuretics [174], and chemotherapeutic drugs [175]. It should be noted that chronic polypharmacy has become normalized in recent decades [176] and so the additive effects of these drugs micronutrient depletion is likely significant.

Rounding out the modern threats to thiamine status in developed countries, pervasive exposures to environmental chemicals and industrial pollutants, damage mitochondrial functioning, even at low, and what are considered, non-toxic exposures [152,177,178,179,180] accelerating the need for thiamine and other mitochondrial nutrients.

## 8. Conclusions

Often thought to be a nutritional issue limited to countries with low and middle income, TD is perceived as being eradicated or anecdotal in high-income countries. Data from a large and growing body of research present a different story; one where frank deficiency may hide behind the guise of common metabolic ailments, and insufficient thiamine is mediated, not by an absence of intake, but by a persistent excess of anti-thiamine exposures. The hyperglycemia-inducing nature of the modern dietary landscape, the regular use of thiamine depleting medications, and exposures to other mitochondrial stressors, make thiamine sufficiency increasingly difficult to maintain in food-secure countries.

As evidenced by the studies presented in Section 4, in food secure countries, TD may present differently than in food insecure populations. It may hide behind more common conditions, be preceded by a long trajectory of marginally insufficient intake relative to need, and present with an extended and varied morbidity. This is consistent with early research where even with severe depletion, where intake was a fraction of the RDA (.15 mg to 45 mg) for an extended period (up to 6 months), so long as calories and other nutrients were maintained to some degree, while morbidity was severe, mortality remained low. During this time, symptomology was non-specific and variable, marked by everything from mood lability, chronic fatigue and muscle weakness, through dysbiosis, dysmotility and food intolerances. It was not until much later, if ever, that the more recognizable symptoms of wet or dry beriberi or WE appeared [4,5,181] These studies found that with sufficient calories, TD presented differently than in the rodent research or in food-insecure countries where both morbidity and mortality are high and align more closely with familiar expectations of TD [64].

In contrast to the linear deprivation of experimental models, starvation, or with acute illness or injury, thiamine inadequacy in the general population in food secure countries is more likely to come with an excess of calories, inducing hyperglycemic cascades and associated illnesses, and may oscillate between periods of sufficiency and deficiency relative to stressors. Across time, repeated decrements to thiamine sufficiency may erode metabolic capacity and flexibility leaving the individual one stressor away from frank and recognizable deficiency. From this perspective, reliance on heavily processed but fortified food products to meet thiamine requirements may precipitate the very deficiencies these products were designed to prevent, while assurances of thiamine sufficiency based upon intake estimates relative to RDA values, likely obfuscate early indicators of a looming crisis.

The reduction in oxidative capacity and the rerouting of glucose, amino and fatty acids through alternate metabolic pathways limits mitochondrial energy capacity, increases ROS, and polyol/sorbitol, hexosamine, diacylglycerol/PKC, AGE pathway associated metabolic toxins, and stabilizes HIF proteins leading to inflammation, immune dysregulation, altered cellular apoptotic pathways. If left unchecked, protracted insufficiency may become TD in the conventional sense, particularly if faced with an acute stressor. More frequently, however, these illnesses present as one or more of the laundry list of chronic ailments associated with poor metabolic capacity. Hyperglycemic-related illnesses are top among them. Given the high rate of metabolic dysfunction observed in western countries, perhaps it is time to redress concepts associated with micronutrient sufficiency and deficiency and reassess diagnostic parameters associated with TD relative to the current dietary and chemical exposure landscape. Future research is needed to expand both the definition and the degrees of thiamine involved illnesses.

## Figures and Tables

**Table 1:** Metabolic effects of thiamine in vivo and in vitro.

In Vivo	
Study	Outcome
Effect of thiamine repletion on cardiac fibrosis and protein O-glycosylation in diabetic cardiomyopathy [138].	In STZ induced diabetic rats, thiamine reduced or reversed hyperglycemia related activation of secondary glucose pathways (polyol/sorbitol, hexosamine, diacylglycerol/PKC, AGE) via upregulation of the PDC enzyme; improved cardiac contractility, reduced cardiac fibrosis and expression of mRNA associated proteins (thrombospondin, fibroconnection, plasminogen activator inhibitor 1, and connective tissue growth factor); and prevented obesity in the overfed arm of the experiment.
Prevention of incipient diabetic nephropathy by high-dose thiamine and benfotiamine [139].	High-dose thiamine and benfotiamine therapy increased TKT and PDC activity in STZ induced diabetic rats, increasing ribose-5-phostphate and reduced microalbuminuria and proteinuria by 70–80%. PKC, AGE and oxidative stress were reduced significantly.
Vitamin B1 analog benfotiamine prevents diabetes-induced diastolic dysfunction and heart failure through Akt/Pim-1–mediated survival pathway [140].	Benfotiamine prevented hyperglycemia induced diastolic dysfunction and heart failure by several mechanisms in STZ induced diabetic rats
Powerful beneficial effects of benfotiamine on cognitive impairment and beta-amyloid deposition in amyloid precursor protein/presenilin-1 transgenic mice [141].	Benfotiamine improved spatial memory, amyloid precursor protein/presenilin-1, reduced amyloid plaques and tau levels dose dependently after 8 weeks of treatment in mouse model.
In Vivo	
Effect of thiamine administration on metabolic profile, cytokines and inflammatory markers in drug-naïve patients with type 2 diabetes [142].	A total of 150 mg thiamine daily significantly reduced blood glucose within a month, in randomized, placebo control trial of 24 drug naïve T2D diabetics
Effect of high dose thiamine therapy on risk factors in type 2 diabetics [68].	A 3 month, randomized, placebo controlled trial of 50 T2D patients, given 100 mg 3x thiamine per day. Thiamine significantly improved micro albuminuria, glycated hemoglobin, while decreasing PKC levels.
The effect of benfotiamine in the therapy of diabetic polyneuropathy [143].	After 45 days of benfotiamine and vitamin B6 supplementation, 19/22 patients saw statically significant reductions in pain, symptom scores, neurophysiological and biological markers of diabetic neuropathy
Metabolic benefits of six-month thiamine supplementation in patients with and without diabetes mellitus type 2 [144].	Six month randomized trial, 60 T2D with medication controlled blood sugar and 26 age- and BMI-matched controls. A total of 100 mg thiamine daily, significantly corrected lipid profiles and creatinine levels.
Thiamine deficiency and cardiovascular disorders [145].	One time administration of 100 mg IV thiamine, improved endothelium-dependent vasodilatation in 10 patients with TD2 during an acute glucose tolerance test.
Thiamine deficiency in patients with congestive heart failure receiving long-term furosemide therapy: a pilot study [146].	A total of 200 mg/day of thiamine for 1 week in 6 patients with HF receiving diuretics improved left ventricular ejection fraction (LVEF) in four of the patients from 24% to 37%.
Thiamine supplementation in symptomatic chronic heart failure: a randomized, double-blind, placebo-controlled, cross-over pilot study [147].	A total of 300 mg/day oral thiamine improved LVEF significantly in HF patients on diuretics.

STZ: streptozotocin; PKC: protein kinase C; T2D: type 2 diabetes; HF: heart failure; LVEF: left ventricular ejection fractions.

## Data Availability

Not Applicable.

## Abbreviations

TD: thiamine deficiency; TPP: thiamine pyrophosphate; TTP: thiamine triphosphate; TMP: thiamine monophosphate; HIF: hypoxia inducible factor; OXPHOS: oxidative phosphorylation; TKT: transketolase; PPP: pentose phosphate pathway; NADPH: nicotinamide adenine dinucleotide phosphate; PDC: pyruvate dehydrogenase complex; AGE: advanced glycation end-product; TCA: tricarboxylic cycle; LDH: lactate dehydrogenase; HACL: 2-hydroxyacyl-CoA lyase; BCAA: branched chain amino acids; BCKAD: branched chain keto-acid dehydrogenase; a-KDGH: alpha-ketoglutarate dehydrogenase complex; ROS; reactive oxygen species; ETC: electron transport chain.
